# Hospital Bring-Your-Own-Device Security Challenges and Solutions: Systematic Review of Gray Literature

**DOI:** 10.2196/18175

**Published:** 2020-06-18

**Authors:** Tafheem Ahmad Wani, Antonette Mendoza, Kathleen Gray

**Affiliations:** 1 School of Computing and Information Systems The University of Melbourne Melbourne Australia; 2 Centre for Digital Transformation of Health The University of Melbourne Melbourne Australia

**Keywords:** BYOD, bring-your-own-device, health care facilities, mhealth, mobile phone, confidentiality, computer security

## Abstract

**Background:**

As familiarity with and convenience of using personal devices in hospitals help improve the productivity, efficiency, and workflow of hospital staff, the health care bring-your-own-device (BYOD) market is growing consistently. However, security concerns owing to the lack of control over the personal mobile devices of staff, which may contain sensitive data such as personal health information of patients, make it one of the biggest health care information technology (IT) challenges for hospital administrations.

**Objective:**

Given that the hospital BYOD security has not been adequately addressed in peer-reviewed literature, the aim of this paper was to identify key security challenges associated with hospital BYOD usage as well as relevant solutions that can cater to the identified issues by reviewing gray literature. Therefore, this research will provide additional practical insights from current BYOD practices.

**Methods:**

A comprehensive gray literature review was conducted, which followed the stepwise guidelines and quality assessment criteria set out by Garousi et al. The searched literature included tier 1 sources such as health care cybersecurity market reports, white papers, guidelines, policies, and frameworks as well as tier 2 sources such as credible and reputed health IT magazines, databases, and news articles. Moreover, a deductive thematic analysis was conducted to organize the findings based on Schlarman’s People Policy Technology model, promoting a holistic understanding of hospitals’ BYOD security issues and solutions.

**Results:**

A total of 51 sources were found to match the designed eligibility criteria. From these studies, several sociotechnical issues were identified. The major challenges identified were the use of devices with insufficient security controls by hospital staff, lack of control or visibility for the management to maintain security requirements, lack of awareness among hospital staff, lack of direction or guidance for BYOD usage, poor user experience, maintenance of legal requirements, shortage of cybersecurity skills, and loss of devices. Although technologies such as mobile device management, unified endpoint management, containerization, and virtual private network allow better BYOD security management in hospitals, policies and people management measures such as strong security culture and staff awareness and training improve staff commitment in protecting hospital data.

**Conclusions:**

The findings suggest that to optimize BYOD security management in hospitals, all 3 dimensions of the security process (people, policy, and technology) need to be given equal emphasis. As the nature of cybersecurity attacks is becoming more complex, all dimensions should work in close alignment with each other. This means that with the modernization of BYOD technology, BYOD strategy, governance, education, and relevant policies and procedures also need to adapt accordingly.

## Introduction

### Background

Bring-your-own-device (BYOD) is a term that refers to the use of personal devices by employees for professional purposes. These devices typically include smartphones, tablets, and laptops and may even include internet of things (IoT) devices such as wearables. Health care is one of the leading industries driving BYOD usage [1-5]. Health care professionals are known to use personal mobile devices for work such as documenting clinical notes; accessing medical records, drug information, or test results; time-tabling; communicating with other staff as well as with patients; and looking up reference resources [1,6-9]. It is suggested that BYOD saves time and improves the productivity of clinicians, makes patient care more efficient through better care coordination and continuity, saves device procurement costs for health care organizations, and may even reduce hospital admission rates [10-12].

However, one of the key issues that BYOD faces is data security. The health care industry sees the greatest number of data breaches among all major industries around the world [13,14]. In part, health care is a target of cybercriminals for various reasons; for instance, medical credentials are said to be sold in the black market, especially the dark web, for over US $1000 [15,16].

One of the primary reasons for health care data breaches is BYOD itself. Hospitals may have little or no control over the security of their employees’ personal mobile devices, which may contain sensitive organizational data such as patient information. Hospitals also do not have any control over a user’s nonwork-related activity on their BYOD device, as ownership lies with the employee. In addition, health care IoT devices such as personal wearables are growing at an exponential rate, and with each device added to the hospital network, the chance of breach increases. Furthermore, given the highly regulated nature of the health care industry, which enforces strict measures to protect patient information, health care organizations face a heavy task of compliance with health data protection laws [17-19]. In short, BYOD security is “one of the biggest headaches for healthcare IT management” [20].

### Objectives

There has been little research into BYOD security, especially in health care [21-23]. Our previous literature review of hospital BYOD security issues and risk mitigation found mostly expert commentaries and a dearth of real-life studies in clinical settings [17]. A limitation of our previous review was that only peer-reviewed literature was considered.

Therefore, the aim of this study was to investigate the challenges and solutions of hospital BYOD security by reviewing the gray literature. The aim of this paper meets the criteria set out by Garousi et al [24] for including gray literature in research: to reduce the gap between academic research and industry practices, to provide perspectives missing from peer-reviewed research, and to provide practical insights about hospitals’ BYOD usage.

## Methods

This gray literature review followed the stepwise guidelines set out by Garousi et al [24].

### Search Process

First, specialized and credible health information technology (IT) sources, which include magazines, databases, and news sources such as Xtelligent Healthcare Media (HITInfrastructure and HealthITSecurity), Pulse IT Communications, Healthcare IT News Australia, and Healthcare Information and Management Systems Society (*HIMSS*) media were searched.

Second, other tier 1 and tier 2 gray literature sources searched from Google and the market research platform *Gartner*, which fit the quality assessment criteria of Garousi et al [24], were considered. Tier 1 sources include highly credible sources where knowledge and authority of the source are well established and where content is produced in conformance with well-defined criteria [25]. These included mobile security white papers and reports, national health care department guidelines or policies, BYOD market research reports, and frameworks from reputable agencies and organizations [24]. Tier 2 sources include sources where knowledge and authority of the source are moderately credible [25]. These included news articles, company annual reports, and document presentations [24]. For these sources, health care terms were used in addition to the terms mentioned above, as part of the search string. These include terms such as “health,” “healthcare,” and “hospital.” Only the top 100 search results from tier 1 and 2 sources were inspected, as saturation of concepts was observed after this.

Finally, some articles were also extracted through *snowballing* of links or citations provided in the abovementioned sources.

### Quality Assessment and Eligibility Criteria

Only articles that fit the quality assessment criteria established by Garousi et al [24] were chosen for the study. Articles were assessed based on the authority of source, method, date, objectivity, novelty, and impact to determine their suitability for this study. Articles from credible and reputable sources (first- and second-tier gray literature), with a clear objective and adding a unique perspective to the research or corroborating previous scientific evidence, were included.

Only gray literature articles published from 2016 to 2019 in the English language were considered eligible. This ensured contemporaneity and practical relevance of this research, given that BYOD security management has seen significant changes during this period, such as an increase in the number and type of data breaches as well as improvements in technology. In addition, this study was limited to mid- and large-size clinical settings, mainly hospitals and community health centers; smaller settings with budget or technological constraints to invest in BYOD security, such as private practices, were excluded. Finally, eligibility was strictly limited to security issues related to the usage of BYOD in hospitals. Issues such as bandwidth, availability, device interference, and medical infection risks were excluded.

**Figure 1:**
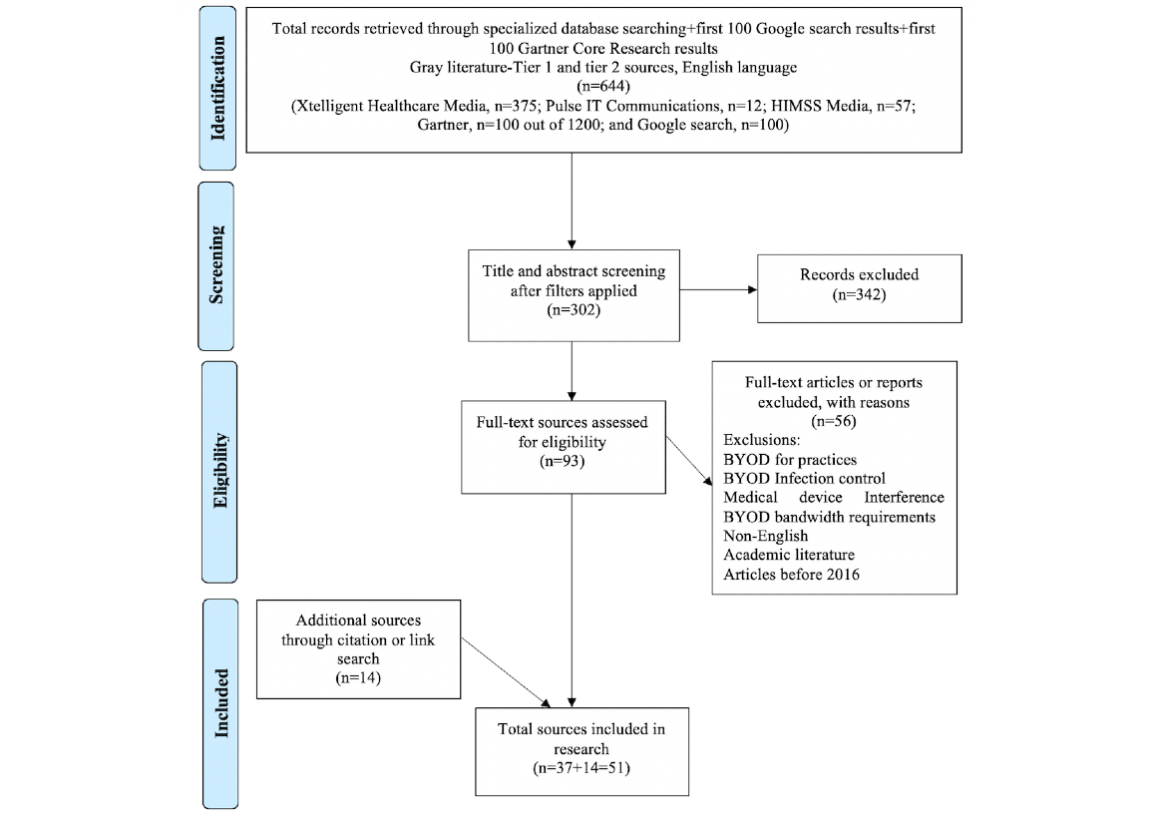
Reporting Items for Systematic Reviews and Meta-Analyses (PRISMA) flowchart.

Overall, 51 articles were included, as shown in the Preferred Reporting Items for Systematic Reviews and Meta-Analyses flowchart [26] in Figure 1.

### Data Extraction and Synthesis

Deductive thematic analysis was conducted using the People Policy Technology (PPT) model by Schlarman [27] to organize the findings [28] in accordance with the previous review conducted by the authors [17]. The PPT model breaks down the security process into 3 core elements: people responsible for executing and supporting the security process; policy used for explaining supporting procedures and providing a clear direction for ideal security behavior; and technology, which includes products, tools, or materials used for supporting the security process [27]. Previous studies indicate that to optimize the cybersecurity controls, the alignment between technical and social dimensions is necessary. Therefore, a holistic approach is required to completely understand the security process [28-31]. Experts also say that the technocentric nature of the present cybersecurity practices has increased the success of cyberattacks as the social dimension of security is relatively neglected [32]. This makes the PPT model very useful as it advocates for equal emphasis on all 3 dimensions (people, policy, and technology) of the security process.

## Results

### Characteristics of the Included Gray Literature

Table 1 summarizes the types of articles included. The detailed characteristics of each article are provided in Multimedia Appendix 1.

**Table 1:** Characteristics of the included gray literature (N=51).

Characteristics		Studies, n (%)	
**Year of publication**			
	2019		7 (14)
	2018		12 (24)
	2017		22 (43)
	2016		10 (20)
**Type of study**			
	Primary		29 (57)
	Secondary		22 (43)
**Outlet source**			
	Market report		12 (24)
	News article		17 (33)
	Opinion post		7 (14)
	Legislation		6 (12)
	White paper		5 (10)
	Policy document		2 (4)
	Newsletter post		2 (4)
**Outlet type**			
	First-tier gray literature		14 (27)
	Second-tier gray literature		37 (73)

### Hospital Bring-Your-Own-Device Security Challenges

Hospitals face several data security challenges owing to the use of BYOD. These challenges are sociotechnical in nature. Therefore, based on the PPT model, this study classifies them as *technology*, *policy*, and *people* challenges [27].

#### Hospital Bring-Your-Own-Device Technology Challenges

The following sections discuss the key technical challenges associated with the use of BYOD in hospitals, which pose a threat to data security.

##### Devices With Insufficient Security Control

Personal mobile devices (BYOD devices) used by health care professionals lack the security controls and visibility of company-owned devices that may have preinstalled security settings and enable better security management [33,34]. For example, in a 2016 survey of US health care organizations, 11% of doctor-owned personal devices that stored patient data had highly vulnerable operating systems that were either outdated or jailbroken/rooted [35]. Although outdated versions may lack the necessary resistance against modern security attacks, jailbroken or rooted devices forcefully block security controls in lieu of additional functionality/control [34].

##### Device Locking/Authentication

According to the same survey, 14% of the devices owned by doctors contained some form of patient data, yet had no device locking or authentication mechanism for protecting sensitive information such as passwords, pattern locks, or biometric authentication [35].

##### App Security

The same survey also revealed that about 27.79 million mobile devices that had installed medical apps were infected with at least one high-risk malware, through downloading vulnerable apps from unregulated app stores [35].

Overall, 46% of the doctors shared patient data through picture messaging, 65% through SMS, and 33% via WhatsApp, according to the mentioned survey [35]. Similarly, 87% of the staff at a National Health Services (NHS)-based hospital in the United Kingdom were found to use such apps to discuss patient cases at work [36]. Consequently, their patients’ health information might be viewed by their colleagues or family members who have access to these platforms [37].

##### Network Security

The Skycure survey also revealed that 39% of the devices used by doctors for their day-to-day practice were susceptible to network attacks by the fourth month of using the device [35], typically when the clinicians connected their devices to unsecure networks such as public hotspots [34,38].

#### Hospital Bring-Your-Own-Device Policy Challenges

The following sections discuss the key BYOD policy-related challenges in hospitals.

##### Lack of Direction

About 62% of health care executives from US health care organizations stated that their hospitals either do not have a BYOD policy or that they are not aware of it [39]. Absence of a dedicated BYOD policy or BYOD program means that there may be a lack of clarity about associated issues such as corporate chain of responsibility, data ownership, data protection, prerequisites for device enrollment, access control, or clinical communication and compliance [33,40].

##### Legislative Compliance

Given the highly regulated nature of the health care industry, health care organizations need to maintain strict compliance with privacy laws. These laws are intended to protect patient privacy and provide transparency to patients in terms of who uses their data and how they are used or transmitted. They make it compulsory for health care organizations to strictly enforce strong and appropriate security controls and also define standard operating procedures. The notification of breaches to the government has also become compulsory under health data privacy laws [41]. Examples of such laws include the Australian Privacy Principles of 1988 and the Healthcare Identifiers Act of 2010 in Australia, the Health Insurance Portability and Accountability Act (HIPAA) and the Health Information Technology for Economic and Clinical Health (HITECH) Act in the United States, the General Data Protection Regulation (GDPR) in the European Union, and the Personal Health Information Protection Act (PHIPA) and the Personal Information Protection and Electronic Documents Act (PIPEDA) in Canada [42-47].

##### Penalties

In case of failure to provide adequate security safeguards, or if found responsible for data breaches, health care organizations may face heavy penalties from the government, in addition to reputational damages. For instance, personal data of 3800 patients at a children’s hospital in Dallas, United States, were accessed from an international airport from an unencrypted, nonpassword-protected Blackberry device, which led to a fine of US $3.2 million over patient privacy breaches [48]. In another example, a lost laptop owned by an employee at a Pennsylvania-based cardiology center led to a breach of 1391 electronic patient records, for which the center was fined US $2.5 million [49].

#### Hospital Bring-Your-Own-Device People Challenges

The following sections discuss the key social or people-related challenges associated with the use of BYOD in hospitals.

##### Inappropriate Behavior

Health care is the only industry where insider threats such as human error and system misuse are more prevalent than external threats such as hacking. Overall, 35% of all insider threats in the health care industry are attributed to human error, whereas 24% of them occur because of system misuse [14]. BYOD is a major contributor to human error as it converges private and organizational data, thus increasing the chance of unintentionally sending patients’ information to the device owner’s friend or family member [37]. System misuse occurs when employees abuse the authority or permissions given to them, for example, retrieving personal information about a patient for purposes not related to health care [14]. BYOD limits the control of hospitals in managing sensitive organizational data and, therefore, increases the chances for system misuse to occur.

##### Lack of Awareness

Employee awareness is a critically important component of any BYOD program [14]. Phishing scams, fake tech support requests, and ransomware attacks have been successfully used in recent times [41,50]. In 2017, HIMSS Analytics revealed that 80% of the surveyed health IT executives rated employee awareness as the foremost concern related to health care data security [50,51].

##### Poor User Experience

If clinicians have to go through complex security procedures such as typing in long passwords or logging in repeatedly after periods of inactivity, they will use work-arounds instead, such as using common or easy-to-remember passwords, sharing passwords with colleagues, or using unauthorized/banned messaging apps such as WhatsApp for communication to minimize lost time, which is again a threat to BYOD security [38,52].

##### Skills Shortage

Overall, 82% of IT executives in a survey of 8 developed countries, including the United States, the United Kingdom, and Australia, said that there is a shortage of cybersecurity skills, and 76% of IT executives believed that their government was not investing sufficiently in cybersecurity talent. In addition, 25% of the respondents claimed that a lack of cybersecurity staff made their organization susceptible to cyberattacks [53].

### Hospital Bring-Your-Own-Device Security Solutions

As discussed, to curb BYOD security risks, a holistic approach is required, with equal emphasis on technology, people, and policy-based measures. This section, therefore, classifies the solutions based on the PPT elements of the security process [27].

#### Hospital Bring-Your-Own-Device Technology Solutions

Technology is one of the core components of the security process, which can aid in efficiently managing BYOD security. The following are important technologies used to curb BYOD security risks.

##### Mobile Device Management

Mobile device management (MDM) is a platform used to manage devices existing within an enterprise centrally. It performs functions such as automation of device registration and deregistration as well as updating or patching of BYOD devices by remotely installing secure configurations, settings, and policies [34]. Moreover, MDM also automates remote installation of enterprise apps such as antivirus or antimalware *over-the-air* onto devices or scans enterprise networks for vulnerabilities [54,55]. Furthermore, it also automates the enforcement of organizational policies such as enabling screen lock or log-off functionalities; encrypting hospital data; securing remote connections through virtual private networks (VPNs); tracking device location; wiping, locking, and securing devices remotely; and whitelisting and blacklisting apps and devices such as jailbroken/rooted devices or unapproved third-party apps [33,56,57].

##### Containerization

Containerization allows logical separation of organizational and personal data. This means that the hospital will only have control over the *container* where the hospital data reside, rather than the whole device. Containers are encrypted and therefore protect sensitive patient data that may reside on the employee’s device. The hospital can scan these data for viruses. It can even lock or delete the data remotely, while keeping the personal data intact [52]. In addition, containers also allow separate cloud backups for both personal and organizational data. Personal data can be uploaded on a personal cloud, whereas hospital data are uploaded on corporate or private cloud. IT administrators retain full control of the containers, and the need to manage the whole device is eliminated [52,58].

##### Virtual Desktop Infrastructure

Virtual desktop infrastructure eliminates BYOD security risks by removing the need to store hospital data on employees’ personal devices. It can provide access to hospital data through remote servers owned by the hospital, which can be connected to via a VPN after logging in remotely with proper credentials [34].

##### Identity and Access Management

Identity and access management (IAM) technologies ensure appropriate access to verified BYOD users through strong authentication, authorization, and access control mechanisms. Modern IAM solutions used in health care typically involve dual-factor authentication. In addition to supplying the username/passwords, users have to use an additional factor for authentication, for example, a biometric such as fingerprint, iris, or face [58]. IAM solutions also provide a feature called *role-based access control*, which ensures that permissions to access or modify patient data depend on the role of a person [34].

In a time-sensitive industry such as health care, the last thing clinicians want is cumbersome or repeated log-ins [38,52]. Single sign-on solutions avoid this, as the user needs to authenticate only once when accessing hospital services, rather than separately authenticating for each hospital app [34,58].

##### Endpoint Security Tools

BYOD devices need to be secured within as well as outside the hospital network. Therefore, endpoint security tools such as antivirus, antimalware, antispyware, or antiphishing tools need to be installed and regularly run on BYOD devices within hospital containers to protect and isolate hospital data within the device [52].

##### Secure Clinical Communication Platforms

Secure health care communication platforms provide an extra layer of security through strong encryption [37,59]. The United States has developed a national encryption standard called *Direct* for secure exchange of health care data, which provides guidelines on safe, scalable, and standards-based clinical communication and also ensures strict compliance to HIPAA [60].

##### Other Emerging Technologies

Several technologies have the potential to revolutionize the BYOD security management process. Unified endpoint management (UEM) is considered to be a good MDM alternative as it provides a single unified interface for managing all types of devices existing within the enterprise, such as PCs, laptops, smartphones, tablets, IoT devices, and wearables, which include both BYOD and company-owned devices. It also allows better methods of managing hospital apps/data, confining them to a secure workspace and separating the personal data of caregivers, which ensures the privacy of both personal health information (PHI) and personal data [61,62].

Another important technology that is gaining adoption is cloud access security broker (CASB), which is used in cloud-based MDM platforms and allows an organization to extend its security policies even outside its infrastructure and therefore manage the organizational data on the device even outside organizational parameters [63]. Health care can significantly benefit from this technology, given the mobile nature of its workforce comprising people who may work with different hospitals or health care organizations. Secure web gateway is another emerging technology that ensures that unsecured traffic, which may be initiated from BYOD devices such as malicious traffic from the web, viruses, or malware, does not enter the internal network of an organization [63]. As these technologies are relatively new, they still have not seen widespread adoption, but organizations are seriously considering their procurement [64].

#### Hospital Bring-Your-Own-Device Policy Solutions

Policy provides the required strategies, rules, and guidelines for the implementation of BYOD. The following policy components form an important part of the BYOD program.

##### Bring-Your-Own-Device Strategy and Governance

Given the lack of direction in hospitals regarding BYOD security, hospitals need to define a comprehensive hospital-wide strategy for BYOD, which should be regularly reviewed and updated. This strategy should be aligned with the hospital’s core values, future vision, and needs and requires close collaboration among all relevant stakeholders, including both clinical and nonclinical staff [65,66]. This strategy must take into consideration previous relevant procedures, data flows, and clinical workflows to understand what hospital data may be stored or transmitted from the clinician’s devices. The hospital must define who will have access to what information and where. It must also ensure that clinical productivity is not hampered [34].

##### User Agreement

Before joining the BYOD program, employees must be asked to sign a BYOD user agreement that elucidates the responsibilities of employees, defines penalties in case of noncompliance, and highlights the responsibilities of the hospital as well. An example of a BYOD user agreement is Queensland Health’s BYOD self-managed service policy document available on the web [67].

##### Bring-Your-Own-Device Policy

Before the rollout of the BYOD program in the hospital, policies that are in line with the BYOD strategy need to be put in place. Important elements that should be included in a BYOD policy are mentioned in Table 2 [33,34,58,65,68].

Health care examples that exhibit these elements include Queensland Health’s BYOD policy document and the sample BYOD policy by the UK NHS [67,69].

**Table 2:** Key elements of a hospital’s bring-your-own-device policy.

Item	Description
Key definitions	Scope, purpose, and governance structure of the BYOD^a^ program, along with the definition of important terms used in the policy.
Service provision	Specifies the process of enrollment, registration, and deregistration.
Access control	Defines who will have access to what information and when. This is particularly important for personal health information, where the principle of least privileges must be applied. Only the required information must be supplied and only when needed, especially when it comes to patient data.
Data storage	Specifies what hospital data are allowed to be stored on BYOD devices and how. If backup is involved, the policy should also advocate for separate backup of personal and hospital data.
Incident reporting	Defines the procedure for reporting cases of breaches, including cases of theft/loss of device. Employees must report such cases to the IT^b^ department, especially if patient data are involved, and the IT department must report it to government agencies in case of major breaches.
Legislation and noncompliance	Defines applicable privacy or health care laws as well as actions or penalties in case of noncompliance with the policy or in case of breaches caused by employee’s personal devices.
Education strategy	Strategies to train employees periodically to ensure secure user behavior. BYOD users should be constantly updated about latest cybersecurity threats. Policies should be disseminated through all means possible. Changes in policies should also be communicated.
Acceptable use	States the purposes for which BYOD devices could be used, whether clinical or nonclinical, and by whom. It defines reasonable use and prohibited activities.

^a^BYOD: bring-your-own-device.

^b^IT: information technology.

#### Hospital Bring-Your-Own-Device People Solutions

People form a critical part of the BYOD security process. The following are important measures that help improve the user security behavior of hospital employees.

##### Security Culture

All groups of hospital employees, which include the hospital’s senior management, the IT department, and BYOD users (both clinical and nonclinical staff), should be actively consulted throughout the duration of the BYOD program and made aware of their responsibilities. The leadership should make security an organizational priority so that clinicians understand the value of preserving the privacy of sensitive patient data. These steps will help in establishing a safe and secure BYOD culture, where the privacy of hospital data is taken seriously [34,65].

##### Employee Awareness and Training

Modes of training can include classroom training, computer-based training, staff meetings, monthly newsletters, posters, and regular team discussions [41]. A study conducted in 6 US health care organizations over 8 years (2011-2018) highlights the effectiveness of antiphishing campaigns in improving the security behavior of health care professionals [50].

##### Skills Improvement

Table 3 provides a summary of hospital BYOD security challenges and solutions.

Experts advocate for government investment in cybersecurity education and research and incorporation of practical training as part of academic programs. Employers also need to support their employees to complete cybersecurity certifications [70].

**Table 3:** Summary of hospital bring-your-own-device security challenges and solutions.

People, policy, and technology dimension and challenges		Solutions
**Technology**		
	Weak authentication mechanisms	Identity and access management/MDM^a^ to manage user authentication centrally Strong passwords Two-factor authentication with single sign-on Automatic log off after periods of inactivity
	Malicious medical apps downloaded on BYOD^b^ devices	Internal/regulated app stores Whitelist/blacklist apps using MDM
	BYOD devices connected to unsecure networks/hotspots	Over-the-air network scanning Remote access through virtual private network Data protection in rest and motion (use of AES^c^/TLS^d^)
	Vulnerable devices connected on hospital network	MDM to prevent vulnerable devices from connecting to hospital networks Network scanning
	Mixing of personal and hospital data	Containerization for logical separation of hospital and personal data Use sandboxed apps for PHI^e^ access Use secure and encrypted clinical communication platforms
	Lost device containing sensitive PHI	Use MDM to track/lock device remotely Use MDM with containerization to selectively wipe hospital data Limit storage of hospital data on device using virtual desktop infrastructure Report theft incidents to hospital information technology department
**Policy**		
	Lack of strategy/direction for ideal BYOD use	Define hospital-wide BYOD strategy to be updated regularly Dedicated BYOD policy for complete guidance on authentication, access control, chain of responsibility, data ownership, devices allowed, acceptable use, training, legislation, and noncompliance Mandating signing of user agreement for BYOD users
	Maintaining compliance with health care data protection laws	Notify relevant government departments about breaches as per law Perform regular audits and legal risk assessments Define applicable privacy regulations and penalties for noncompliance Train BYOD users about incident reporting to notify breaches/thefts
	Access privilege abuse	Use principle of least privileges and role-based access control in defining staff access to PHI
**People**		
	Inappropriate behavior by BYOD users	Penalize staff found guilty of breaches Encourage safe and secure use by establishing a security culture Monitor user behavior regularly
	Lack of awareness among hospital BYOD users	Educate BYOD users periodically Check awareness levels regularly, for example, through phishing campaigns
	Poor user experience	Consult all relevant stakeholders throughout the BYOD program Carefully consider clinical workflow and ease of use
	Cybersecurity budget and skills shortage	Government investment in technology, education, and research Hiring experts Sponsoring and supporting employees for skills improvement

^a^MDM: mobile device management.

^b^BYOD: bring-your-own-device.

^c^AES: Advanced Encryption Standard.

^d^TLS: Transport Layer Security.

^e^PHI: personal health information.

## Discussion

### Principal Findings

A wide range of technological solutions, policy control measures, and social practices are available that can be used together to curb BYOD security risks in hospitals. The findings suggest that the key challenge lies in ensuring a proper balance between usability and security. Therefore, BYOD security management should not only involve the use of resilient security mechanisms but also ensure that the mobility and productivity of hospital employees, especially clinicians, are not hampered. Hospital-owned enterprise productivity apps such as secure messaging and photography or the use of single sign-on for accessing hospital apps can help to address such concerns.

From a technological perspective, the BYOD landscape is changing. Gartner analysts predict that the BYOD model will change to a bring-your-own-environment model as users bring not only devices and apps but also services, personal networks, and even collaborative workspaces beyond email or messaging [71]. It is expected that large health care organizations will have to deal with as many as 80,000 connected IoT devices [72]. Modern BYOD security technologies such as UEM or containerization allow BYOD security management to become device independent. The highlight of these technologies is that they allow logical separation of hospital and personal data on employees’ devices. As such, BYOD security management is moving from a traditional device-centric approach to a data-centric or app-centric approach, to gain complete control of hospital data residing on employees’ devices and relinquish the management of the whole device itself.

On the basis of our findings from the policy perspective, this study highlights how a lack of clarity with regard to optimal BYOD usage prevalent in hospitals can be addressed through a dedicated BYOD policy as it will provide complete direction and guidelines for safe, secure, and productive BYOD usage in the hospital. Important components that must be covered in the policy include governance of the BYOD program, choosing the right technologies to support the program, providing guidelines for appropriate use, training and education strategies for employees, and compliance with health care data privacy laws. In addition, the policy needs to be contextual and inclusive of various stakeholders. The policy must also be in alignment with the organization’s goals and the limits of the hospital’s capabilities. Finally, with rapid changes in BYOD technology, modernization of policy is also required. This means that the introduction or reformation of security standards or protocols needs to be contemplated by considering both security and the clinical workflow [73].

From people’s perspective, effective implementation of technology and policy will require strong consultations with all relevant stakeholders from the management side, which includes the hospital’s senior management; the IT department; and the user side, including BYOD users such as clinicians, administrative staff, and other staff. Educating and training employees about BYOD threats and security measures should be directed to improve their commitment to protect hospital data, especially PHI. The changing BYOD landscape also demands expansion and refreshment of skill set for the management, IT department, and BYOD users. Hospitals may need to recruit external staff capable of successfully implementing complex technologies such as UEM and CASB, which may also require training of hospital IT personnel [62].

### Limitations

As far as the limitations of this study are concerned, the complex nature of gray literature searches means that the number of items available for review is significantly higher than that of peer-reviewed literature searches; therefore, limiting the items analyzed was unavoidable [24]. Nevertheless, some important sources may have been neglected. Furthermore, although some gray literature sources gave examples from real-life studies, others were based on the opinions of credible experts who wrote blogs for well-known media publications. Although these sources are useful, they do not meet the criteria of the highest quality sources.

### Comparison With Prior Work

The use of the PPT model [27] to answer the research questions has aided in providing a holistic perspective of BYOD security management, which may have been lacking in previous studies, as highlighted earlier. This study, therefore, complements and augments the authors’ previous findings, where the same model was used for analyzing peer-reviewed literature [17]. The practical, real-life evidence extracted through the gray literature review not only corroborates the previous outcomes but also provides additional insights.

### Conclusions

As modern BYOD security threats grow in size and complexity, this study elucidates how health care organizations can use technological solutions, policy control mechanisms, and people management measures in close alignment to curb such risks effectively and holistically. This has become very important as cybersecurity is seen as one of the biggest challenges in the health care industry [74], with BYOD being one of the major threats to cybersecurity itself [39,75].

## Appendix

Multimedia Appendix 1 Gray literature summary and characteristics.

## Abbreviations

BYOD: bring-your-own-device
CASB: cloud access security broker
HIMSS: Healthcare Information and Management Systems Society
HIPAA: Health Insurance Portability and Accountability Act
IAM: identity and access management
IoT: internet of things
IT: information technology
MDM: mobile device management
NHS: National Health Services
PHI: personal health information
PPT: People Policy Technology
UEM: unified endpoint management
VPN: virtual private network
