# Supplementary material for: Hospital Bring-Your-Own-Device Security Challenges and Solutions: Systematic Review of Gray Literature
Source: JMIR Mhealth Uhealth. 2020 Jun 18;8(6):e18175. doi: 10.2196/18175 (PMC7333072; doi:10.2196/18175)
Supplement: Multimedia Appendix 1 [file mhealth_v8i6e18175_app1.pdf]

APPENDIX A

S.NO.	AUTHOR/ YEAR	TITLE	Type of GL	OUTLET SOURCE	OUTLET TYPE	BRIEF DESCRIPTION, RELEVANCE AND OBJECTIVITY	Theme	AUTHORITY/ CREDIBILITY EVIDENCE
**1**	Lovell (2019)	WhatsApp use in the NHS a 'privacy and clinical safety timebomb'	Secondary	News Article	2nd tier GL	Based on a study conducted by St George's University Hospital NHS Foundation Trust, it discusses use of consumer messaging platforms in hospitals affiliated to the NHS as it leads to a serious breach in confidentiality. The article proposes use of healthcare data privacy law compliant messaging platforms such as Hospify, Forward and MedxNote.	Clinical communication /messaging (Technology)	MobiHealthNews is a leading publisher of health IT news which is owned by HIMSS media.
**2**	Davis (2019)	Phishing Education, Training Can Reduce Healthcare Cyber Risk	Secondary	News Article	2nd tier GL	Discusses how increased awareness among hospital employees leads to improvement in user security behaviour, which can reduce security breaches. An example of a phishing campaign across US hospitals is given, by quoting a Journal of American Medical Association (JAMA) study, where systematic phishing campaigns led to decrease in click rates.	User awareness/ Training (People)	HealthITSecurity.com is a leading source which publishes news and interviews about cybersecurity, data privacy and compliance in healthcare.
**3**	Spannbauer (2019)	How can healthcare organizations remedy their cybersecurity ailments?	Secondary	Opinion Post	2nd tier GL	In the light of data breach report published by Verizon, the author discusses how healthcare is the only industry, where breaches due to internal threats are more prevalent then external threats, with BYOD being a leading cause as it blurs the lines between personal and professional data. The author also lights the importance of measures such as encrypted communication platforms, strong policies and awareness campaigns in curbing such risks.	General Issues/Solutions (All)	The author is a Senior Director of Product Management at a leading organisation. The analysis is posted on HelpNetSecurity, a reputable information security independent site known for publishing feature articles, expert analysis, reviews, industry news, whitepapers and newsletters.
**4**	Mobileron (2019)	Mobile First Healthcare	Primary	White Paper	1st tier GL	Discusses how Mobileron's Unified Endpoint Management can be used in healthcare to secure patient data on personal mobile devices of clinicians yet preserving clinician mobility. Moreover, the whitepaper expands on how UEM can be used to confine clinical apps to a workspace managed by the hospital, therefore keeping hospital data and personal data separate.	Mobility Management (Technology)	Mobileron is a leading mobile security firm which develops security technologies such as UEM, MDM and Mobile Threat Deference. Mobileron was named as a leader in Gartner's magic quadrant for UEM.
**5**	Bullock (2019)	The Future Of BYOD: Statistics, Predictions And Best Practices To	Secondary	Opinion Post	2nd tier GL	Discusses present trends, future predictions and best practice measures for a future secure BYOD which are necessary to curb modern cybersecurity threats.	Market Trends	Forbes is a leading, reputable business magazine which publishes articles in diverse areas including technology.

		Prep For The Future						
**6**	European Data Protection Supervisor (2019)	GDPR Health Data Protection	Primary	Government legislation	1st tier GL	As a part of the GDPR compliance, the source discusses specific methods of protection for PHI and also defines the rules, regulations and responsibilities of anyone dealing with such data, particularly healthcare providers.	Law, Standard or Policy (Policy)	European Union government legislation concerning personal health data protection published on government website.
**7**	Personal Health Information Protection Act of 2004 (2019)	Personal Health Information Protection Act of 2004	Primary	Government legislation	1st tier GL	The Canadian legislation provides an in-depth interpretation of rules, regulations and responsibilities of relevant parties in protecting the confidentiality, integrity and availability of personal health information.	Law, Standard or Policy (Policy)	Canadian Government legislation concerning personal health data protection published on government website.
**8**	HealthITSecurity (2018)	Developing a Successful, Sustainable Mobile Device Management Program for Healthcare	Secondary	News Article	2nd tier GL	Discusses how MDM can be used to mitigate BYOD security threats through measures such as automatic locking, remote wipe, enforcing authentication, remote updates, data encryption and securing wi-fi as well as VPNs.	Mobility Management (Technology)	HealthITSecurity.com is a leading source which publishes news and interviews about cybersecurity, data privacy and compliance in healthcare.
**9**	Lenovo Australia (2018)	BYOD Trends: Looking to the Future	Secondary	Opinion Post	2nd tier GL	Discusses future outlook of BYOD in the wake of market trends and explains the importance of strong and flexible policies in mitigating future cybersecurity threats.	Market Trends	Lenovo is a reputable technology firm which develops computer products and IT management software.
**10**	Minion (2018)	Healthcare suffers almost a quarter of data breaches, as reports skyrocket under mandatory notification scheme	Secondary	News Article	2nd tier GL	Discusses OAIC's (Office of the Australian Information Commissioner) report on data breaches in the year 2018, where healthcare was found to be the leading industry in Australia affected by data breaches (24%).	Breach Analysis (Market Trends)	HealthcareITNews is an authoritative Australian news source which publishes insightful articles on health IT. It is a member of the HIMSS series of publications.
**11**	Kleyman (2018)	4 Key Ways to Overcome Healthcare BYOD	Secondary	News Article	2nd tier GL	Discusses four key strategies in overcoming healthcare BYOD security challenges. These include a strong, well-documented BYOD strategy, a robust Enterprise Mobility/Mobile Device	General Issues/Solutions (All)	HealthITSecurity.com is a leading source which publishes news and interviews about cybersecurity, data privacy and compliance in healthcare.

		Security Challenges				Management solution, enabling network visibility and complete data scanning and use of secure collaborative tools.		
**12**	U.S. Department of Health & Human Services (2018)	HIPAA Privacy Rule | HITECH Act	Primary	Government legislation	1st tier GL	US-based health data privacy legislations such as HIPAA and HITECH are provided, which explain rules and regulations that protect personal health information and also states patient rights regarding the privacy of their data.	Law, Standard or Policy (Policy)	US Government legislation concerning personal health data protection published on government website.
**13**	Healthcare Executive Group (2018)	Healthcare Executives Rank the Top 10 for 2019	Primary	Market Report	2nd tier GL	Discusses the top ten challenges for healthcare executives for the year 2019, where cybersecurity is seen as a major challenge in the wake of recent attacks.	Market Trends	HealthCare Executive Group is a well-known US-based national network of leaders in the healthcare industry who meet annually to discuss the top ten opportunities, challenges and issues their organisations face.
**14**	SPOK (2018)	Eighth Annual Spok Survey Mobile Strategies in Healthcare Results Revealed.	Primary	Market Report	2nd tier GL	Discusses major outcomes of an annual BYOD survey where leading usage trends, drivers, challenges and security practices are analysed.	Market Trends	SPOK is one of the leading providers of clinical communication, collaboration, mobility and productivity tools. SPOK conducts surveys on mobile device strategy in healthcare organisations annually.
**15**	Verizon (2018)	2018 Data Breach Investigations Report	Primary	Market Report	2nd tier GL	Provides an in-depth investigation report on data breaches reported globally across all verticals, including healthcare. Discusses how and why healthcare is the only industry where internal actors cause more breaches than external threats. Provides actionable insights into various types of breaches.	Breach Analysis (All)	Verizon is one of the leading communication technologies firm which publishes an independent data breach report annually.
**16**	Taylor, Silver, Troni, & Kleynhans (2018)	Hype Cycle for Mobile, Endpoint and Enterprise Wearable Computing, 2018	Primary	Market Report	2nd tier GL	A hype cycle is provided which visualizes the growth, maturity and adoption rate of endpoint and wearable computing platforms in different scenarios, including BYOD.	Market Trends	Gartner is a global market research and advisory firm recognised worldwide for its evidence-based and insightful research.
**17**	Office of the Australian Information Commissi	Australian Privacy Principles	Primary	Government legislation	1st tier GL	Discusses the thirteen important principles that govern the rules, regulations and standards for personal data protection (including health data), based on Australian legislation known as Privacy Act of 1988.	Law, Standard or Policy (Policy)	Australian government legislation concerning personal data protection published on government website.

	oner (2018)							
**18**	Office of the Privacy Commissioner of Canada (2018)	PIPEDA legislation and related regulations	Primary	Government legislation	1st tier GL	Personal Information Protection and Electronic Documents Act (PIPEDA) is a federal Canadian law which establishes rules, regulations and standards regarding personal data protection and their secure handling by businesses.	Law, Standard or Policy (Policy)	Canadian government legislation concerning personal data protection published on government website.
**19**	Bryant (2018)	BYOD use is on the rise, and hospital policies need to be robust	Secondary	Opinion Post	2nd tier GL	Discusses need for important security measures in the wake of increase in BYOD use in healthcare. These include use of a strong, well-documented and flexible BYOD policy, optimal use of MDM platforms to enforce security controls and providing regular training to employees.	General Issues/Solutions (All)	Healthcare Dive is a reputable online news platform known to cover topics such as health IT, policy and regulation and value-based care. The author is one of the leading contributors to the publication.
**20**	O'Dowd (2017)	Healthcare BYOD Security Embraces Cloud, Biometric Authentication	Secondary	News Article	2nd tier GL	Focuses on increase in adoption of cloud security and biometric authentication technologies with increase in BYOD use across healthcare institutes.	Identity and Access Management (Technology)	HITInfrastructure is a part of the same media group as healthITsecurity, which is known to publish news and subject matter expert interviews in the health IT industry.
**21**	Bitglass (2017)	One in Four Organizations Rely Solely on User-Generated Passwords to Secure BYOD Access	Primary	Market Report	2nd tier GL	In the light of a survey conducted at a conference, discusses lack of adequate authentication mechanisms on BYOD devices which increases the risk of unauthorised access.	Identity and Access Management (Technology)	BitGlass is one of the leading vendors of identity and access management solutions regularly publishes market research reports.
**22**	HIMSS (2017)	9 Bring Your Own Device (BYOD) Challenges	Primary	Newsletter Post	2nd tier GL	Discusses the nine key healthcare BYOD challenges which includes lack of a well-defined policy and a chain of responsibility, internal and external data breaches, data backup and recovery, lost mobile devices, lack of passwords, reactive security posture, depth in security mechanisms and change management.	General Issues/Solutions (All)	HIMSS is a global, non-profit and one of the leading health IT societies in the word which regularly publishes articles through its media platform known as 'HIMSS Media'.
**23**	Snell (2017)	4 Key Concerns in Healthcare Mobile Security Options	Secondary	News Article	2nd tier GL	Discusses important decision-making concerns for healthcare IT executives which includes choosing the right technologies for BYOD security and their budgeting, user awareness and	General Issues/Solutions (All)	HealthITSecurity.com is a leading source which publishes news and interviews about cybersecurity, data privacy and compliance in healthcare.

						training strategy as well as understanding healthcare law compliance for effective implementation of BYOD strategy.		
**24**	Schiff (2017)	The 4 biggest healthcare IT headaches	Secondary	Opinion Magazine Post	2nd tier GL	By interviewing leading health IT executives, the source discusses why BYOD is seen as one of the top four challenges by healthcare CIOs. Also proposes use of technologies such as mobile containers, two-factor authentication, offline pin, and selective wiping for effective BYOD security management.	General Issues and Solutions (All)	CIO is a leading technology and IT digital magazine targeted at technology executives and decision-makers.
**25**	Sulleyman (2017)	Why hackers just launched a huge cyber-attack on the NHS	Secondary	News Article	2nd tier GL	In the wake of cyber-attacks on NHS, discusses why healthcare data is extensively targeted by hackers as it can value over ten times the value of financial data on the dark web.	Breach Analysis (All)	Independent is a reputed British online newspaper which pitches itself as an independent news platform.
**26**	Department of Health and Human Services (2017)	$2.5 million settlement shows that not understanding HIPAA requirements creates risk	Primary	Govt. News Article	1st tier GL	Reports HIPAA non-compliance of a cardiology-based institute in US, where a lost laptop led to breach of 1391 patient records and was therefore fined 2.5 million$ due to lack of adequate security measures.	Law, Standard or Policy (Policy)	Published directly by the media wing of the Department of Health and Human Services (HHS), USA.
**27**	O'Dowd (2017)	IoT, BYOD Prompt Healthcare Cloud Security Market Growth	Secondary	News Article	2nd tier GL	Discusses growth in the cloud security market as adoption in BYOD and healthcare IoT increases. Also highlights the need for cloud platforms to securely host clinical applications and therefore be compliant with healthcare privacy laws.	Market Trends	HITInfrastructure is a part of the same media group as healthITsecurity, which is known to publish news and subject matter expert interviews in the health IT industry.
**28**	Snell (2017)	Employee Healthcare Data Security Awareness Top Industry Threat	Secondary	News Article	2nd tier GL	Discusses how employee errors are the leading contributors of healthcare data breaches which is why health IT executives see lack of awareness as the leading threat to data security.	User awareness/ Training (People)	HealthITSecurity.com is a leading source which publishes news and interviews about cybersecurity, data privacy and compliance in healthcare.
**29**	Snell (2017)	Mobile Security Essential Healthcare Provider Priority	Secondary	News Article	2nd tier GL	Discusses BYOD usage trends in healthcare settings in terms of type of mobile device and applications used.	Market Trends	HealthITSecurity.com is a leading source which publishes news and interviews about cybersecurity, data privacy and compliance in healthcare.
**30**	Rice (2017)	Children's Dallas docked $3.2 million over patient privacy breaches	Secondary	News Article	2nd tier GL	Reports a major patient privacy breach in a Children's hospital in the US caused due to a stolen laptop which led to a fine of USD 3.2 million.	Law, Standard or Policy (Policy)	Dallas Morning News is a leading, prize-winning newspaper daily published from Dallas, Texas.

**31**	SPOK (2017)	The State of Mobile Communications in Healthcare: Devices, Infrastructure, And Access-Survey Results	Primary	Market Report	2nd tier GL	Major outcomes of an annual mobile communications survey are reported where healthcare BYOD usage trends, security practices, challenges and opportunities are discussed.	Market Trends	SPOK is one of the leading providers of clinical communication, collaboration, mobility and productivity tools. SPOK conducts surveys on mobile device strategy in healthcare organisations annually.
**32**	ClearData (2017)	Securing Health Data in a BYOD World	Primary	White Paper	1st tier GL	Discusses impact of BYOD usage in healthcare, its top drivers, security risks as well as five key strategies to curb BYOD security risks in a healthcare environment, which includes a combination of technology and policy-based measures.	General Issues/Solutions (All)	ClearData is a reputed US-based organisation which provides healthcare cloud computing solutions which are HIPAA compliant.
**33**	HIMSS Media (2017)	Navigating BYOD and BYOA in healthcare without compromising security	Primary	White Paper	1st tier GL	Discusses benefits of BYOD in improving patient care, various types of security violations caused by BYOD in healthcare settings, mitigation healthcare BYOD security risks and the need to maintain the balance between security and usability for effective BYOD implementation.	General Issues/Solutions (All)	HIMSS is a global, non-profit and one of the leading health IT societies in the word which regularly publishes articles through its media platform known as 'HIMSS Media'.
**34**	Department of Digital Policy and Governance - eHealth Queensland (2017)	BYOD Self-managed service	Primary	Government Policy Document	1st tier GL	A detailed policy document which specifies optimal BYOD use in Queensland hospitals which includes enrolment prerequisites for BYOD devices, security setup, user responsibilities, admin responsibilities, recordkeeping as well as relevant legislations.	Law, Standard or Policy (Policy)	The source is an active policy document which is available online and authored by Department of Digital Policy and Governance - eHealth Queensland.
**35**	HIMSS (2017)	2017 Essentials Brief: Mobile - HIMSS Analytics survey	Primary	Market Report	2nd tier GL	Provides an overview of various mobile technologies used in US hospitals and highlights use of BYOD and its security implications.	Market Trends	HIMSS is a global, non-profit and one of the leading health IT societies in the word which regularly publishes articles through its media platform known as 'HIMSS Media'.
**36**	HIMSS (2017)	20 Questions to Ask About Bring Your Own Device (BYOD)	Primary	Newsletter post	2nd tier GL	Discusses important questions to consider when developing a hospital BYOD strategy. It includes questions for three groups – users, IT Department and management.	Law, Policy or Standard (Policy)	HIMSS is a global, non-profit and one of the leading health IT societies in the word which regularly publishes articles through its media platform known as 'HIMSS Media'.

**37**	Vocera (2017)	7 Tips for Secure BYOD in Healthcare	Primary	Opinion Post	2nd tier GL	Discusses seven important tips for a secure healthcare BYOD program which includes development of a clear and coherent BYOD policy, educating users, biometric authentication, single sign-on, securing applications and blacklisting containers or personally-owned applications on hospital networks.	General Issues/Solutions (All)	Vocera is a reputed US-based company which develops healthcare communication solutions.
**38**	Smith et al. (2017)	Predicts 2018: Mobile, Endpoint and Wearable Computing Strategies	Primary	Market Report	2nd tier GL	A comprehensive market prediction for mobile, endpoint and wearable technologies is provided till 2022 which includes BYOD. Discusses market insights and implementation recommendations.	Market Trends	Gartner is a global market research and advisory firm recognised worldwide for its evidence-based and insightful research.
**39**	Heathcote (2017)	Bring Your Own Device Security: Example Policy	Primary	Government Policy Document	1st tier GL	As a template, provides a sample BYOD policy for UK healthcare organisations. Touches upon important aspects related to BYOD security such as authorisation, acceptable use, user and device authentication, protection of data at rest, protection of data in transit, device patching and updates, device application management, malicious code protection and incident response.	Law, Policy or Standard (Policy)	The source is a sample BYOD policy template directly authored by NHS digital UK.
**40**	Devine (2017)	Bring your own device (BYOD) reality – and what to do about it	Secondary	Opinion Post	2nd tier GL	Discusses important steps in developing a healthcare BYOD strategy which includes conducting a baseline assessment, developing data governance and acceptable-use policy, implementing security provisions that balance security and employee privacy, implementing state-of-the-art secure texting solutions, anticipating device theft or loss and improving user-awareness.	General Issues/Solutions (All)	Becker Hospital Review is an established media platform in the healthcare industry targeted at leaders and decision makers. The author is an experienced leader in healthcare technology and serves as the managing director of Huron, a global consulting group.
**41**	*Healthcare Identifiers Act 2010* (2017)	Healthcare Identifiers Act 2010	Primary	Government legislation	1st tier GL	The legislation establishes rules and regulations for the verification, collection, use and disclosure of healthcare identification information in a secure manner.	Law, Policy or Standard (Policy)	Australian government federal legislation concerning healthcare identification published on government website.
**42**	Hexa Research (2016)	BYOD (Bring Your Own Device) Market Analysis, Market Size, Application Analysis, Regional Outlook,	Primary	Market Report	2nd tier GL	Provides an in-depth market analysis and forecast of BYOD which includes regional outlook, vertical analysis and competitive strategies from the year 2016-2024.	Market Trends	Hexa Research is an established market research and consulting firm, known for providing insightful industry research and consulting services to organisations across the globe.

		Competitive Strategies and Forecasts, 2016 To 2024						
**43**	Snell (2016)	Enabling Providers to Use Truly HIPAA Compliant Email	Secondary	News Article	2nd tier GL	Explains importance of using a fully HIPAA compliant communication or secure messaging platform in healthcare settings and also discusses its usage in the light of surveys conducted in US-based hospitals.	Clinical communication /messaging (Technology)	HealthITSecurity.com is a leading source which publishes news and interviews about cybersecurity, data privacy and compliance in healthcare.
**44**	O'Dowd (2016)	Why the Difference Between MDM, EMM Matters in Health IT	Secondary	News Article	2nd tier GL	Discusses difference between Mobile Device Management (MDM) and Enterprise Mobility Management (EMM) and their relevance in a healthcare context.	Mobility Management (Technology)	HITInfrastructure is a part of the same media group as healthITsecurity, which is known to publish news and subject matter expert interviews in the health IT industry.
**45**	Markets and Markets (2016)	Bring your own device (BYOD) and Enterprise Mobility Market Research Report 2021	Primary	Market Report	2nd tier GL	Discusses trends and forecasts in BYOD and enterprise mobility markets by software, vertical, region, deployment model, software and security.	Market Trends	Markets and Markets is a well-known market research platform which regularly publishes industry insights and market intelligence reports.
**46**	Snell (2016)	Is There a Healthcare Cybersecurity Skills Shortage?	Secondary	News Article	2nd tier GL	Discusses the effects of cybersecurity skills shortage on healthcare organisations, government investments as well as the effectiveness of cybersecurity education and training programs.	Skills shortage (People)	HealthITSecurity.com is a leading source which publishes news and interviews about cybersecurity, data privacy and compliance in healthcare.
**47**	HealthITSecurity (2016)	BYOD Security in the Healthcare Setting	Secondary	News Article	2nd tier GL	Discusses the benefits of BYOD in a healthcare setting, potential mobile security threats that arise due to BYOD usage in healthcare as well as the importance of HIPAA-compliant secure messaging/texting platforms for secure BYOD implementation.	General Issues and Solutions (All)	HealthITSecurity.com is a leading source which publishes news and interviews about cybersecurity, data privacy and compliance in healthcare.
**48**	Skycure (2016)	Mobile Security Trends in Healthcare	Primary	Market Report	2nd tier GL	Discusses key mobile security trends in healthcare as per a survey conducted in US-based healthcare institutions. These include BYOD usage trends among doctors and security vulnerabilities of healthcare BYOD devices.	Market Trends	Skycure was an established mobile threat defense solution provider which was acquired by Symantec in 2017.
**49**	Optum (2016)	Security Implications of BYOD in Health Care	Primary	White Paper	1st tier GL	Discusses impacts of BYOD usage in healthcare, gaps in healthcare BYOD security as well as recommended best-practices for ideal BYOD usage in healthcare settings.	General Issues/Solutions (All)	Optum is a global care services group who has published this whitepaper in collaboration with 'TechTarget' media, a reputed media group which regularly publishes technology-based articles across different industries.

**50**	McAfee (2016)	Hacking the Skills Shortage Report- A study of the international shortage in cybersecurity skills	Primary	Market Report	2nd tier GL	Based on a survey conducted in eight developed countries, discusses cybersecurity skills shortage, its underlying causes its impact on organisations in addressing security issues.	Skills shortage (People)	McAfee is one of the largest global IT security software company owned by Intel.
**51**	Malkary (2016)	"Bring Your Own Device" (BYOD) Initiatives Enable Clinical Transformation	Primary	White Paper	1st tier GL	Discusses how modern mobile technologies will transform BYOD usage in healthcare institutions and streamline clinical workflow. Also explains its implication on security of PHI and the importance of network management technologies in eliminating or mitigating BYOD security risks.	Network Management (Technology)	Author is the managing director of SpyGlass consulting group, a reputed market advisory company.
